# Méthotrexate et psoriasis: à propos de 46 cas

**DOI:** 10.11604/pamj.2014.19.84.5165

**Published:** 2014-09-25

**Authors:** Kawtar Inani, Mariame Meziane, Fatimazahra Mernissi

**Affiliations:** 1 Service de Dermatologie, CHU Hassan II, Fès, Maroc

**Keywords:** Méthotrexate, psoriasis, MTX, Methotrexate, psoriasis, MTX

## Abstract

Le psoriasis est une maladie inflammatoire chronique, son traitement peut être local ou général. Le méthotrexate (MTX) est parmi les traitements systémiques du psoriasis modéré à sévère. Le but de notre étude est d’évaluer la place du MTX dans le traitement du psoriasis dans notre contexte marocain. C'est une étude rétrospective menée au service de dermatologie du CHU HASSAN II FES de 2010 à 2013. 46 patients ont répondus aux critères d'inclusions. Il s'agissait de patients de sexe masculin dans 58,7% des cas, de sujets âgés entre 18 et 45 ans dans 45,7% des cas. Le psoriasis vulgaire était la forme la plus répondue (76,1%), 56,5% avaient une surface corporelle(SC) atteinte comprise entre 25 et 50%, L’évolution était marquée par une rémission complète dans 50% des cas. Le MTX a été utilisé depuis plus de 40 ans dans le traitement du psoriasis modéré à sévère. Dans notre série le recours au MTX était nécessaire et ceci après échec d'autres thérapeutiques. Son efficacité a été constatée chez 50% des patients, avec peu d'effets secondaires. Le MTX est une molécule de référence dans le traitement du psoriasis modéré à sévère, avec un meilleur rapport coût/bénéfice/risque.

## Introduction

Le psoriasis est une maladie inflammatoire chronique caractérisée par une activation anormale du système immunitaire affectant principalement la peau et les articulations. Le début est précoce, généralement entre 20 et 30 ans. Le psoriasis a un impact majeur sur la qualité de vie des patients. Ces derniers sont confrontés à des dépenses personnelles importantes, et à l′exclusion sociale [1]. Sa prise en charge est un vrai challenge vu la chronicité de la pathologie chose qui nécessite une bonne observance, et vu le cout élevé des traitements et leur non disponibilité. Le méthotrexate (MTX) est un analogue de l′acide folique, qui agit comme un agent antinéoplasique et anti-métabolique en inhibant la prolifération cellulaire. Il a été utilisé depuis les années cinquante, dans plusieurs domaines notamment en médecine interne, en rhumatologie et en dermatologie. Actuellement, Il constitue le traitement systémique de référence du psoriasis cutané modéré à sévère [2, 3]. L'objectif de notre étude est d’évalué l'efficacité, la tolérance du MTX dans le traitement du psoriasis sévère à modéré ainsi que l'identification des effets secondaires chez nos patients.

## Méthodes

Il s'agissait d'une étude rétro-prospective uni centrique avec un recueil de données des patients psoriasiques suivis au service de Dermatologie du CHU Hassan II de Fès de 2010 à 2014. Les patients incluent dans l’étude avaient une surface corporelle > 25% et n'avaient aucune contre indication absolu à un traitement par méthotrexate. Tous les cas de psoriasis ont été inclus et les dossiers des patients traités par MTX ont été étudiés. 200 cas de psoriasis ont été colligés dont 46 avaient une surface corporelle (SC) > 25% et n'avaient aucune contre indication absolu à un traitement par méthotrexate.

Une fiche préétablie a été remplie pour chaque patient précisant les données socio-épidémiologiques (l’âge, le sexe, le Numéro de téléphone, la date de début de psoriasis (ancienneté), et les traitements prescrits),les données cliniques (recueillies grâce à un examen clinique municieux de la peau, des muqueuses et des phanères, afin de déterminer le type de psoriasis, la SC était mesurée soit par la règle de Wallas soit par la paume de la main),les données Thérapeutiques, ainsi ont été précisées ( la dose d'attaque du MTX, la voie d'administration, la date du début traitement, les effets secondaires, la dose cumulée, la supplémentation ou non en acide folique, et l'association à d'autres traitements locaux), les données para cliniques (Bilan biologique et radiologique initiaux et de control: NFS, bilan hépatique, bilan rénal, albumine, bilan infectieux, hormone gonadotrophine chorionique, et fibroscann) et enfin les données évolutives, on a considéré comme: -rémission complète: la guérison complète des lésions à l'arrêt du MTX; - Amélioration: une diminution de la surface corporelle atteinte de 20% Les données étaient saisies et validées sur un fichier Excel pour être analysées à l'aide du logiciel SPSS20.

## Résultats

**Figure 1:**
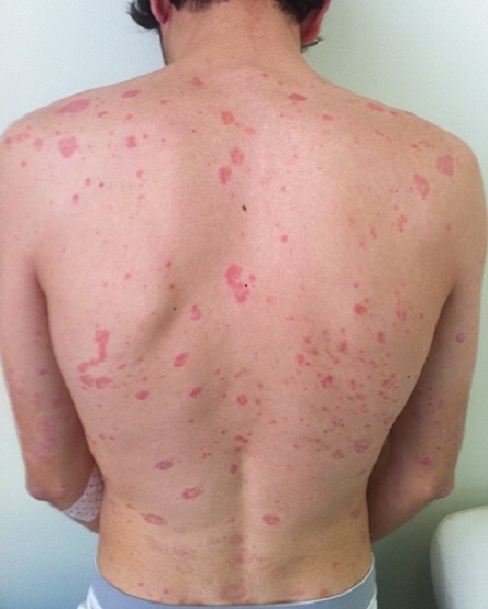
Psoriasis en plaque

**Figure 2:**
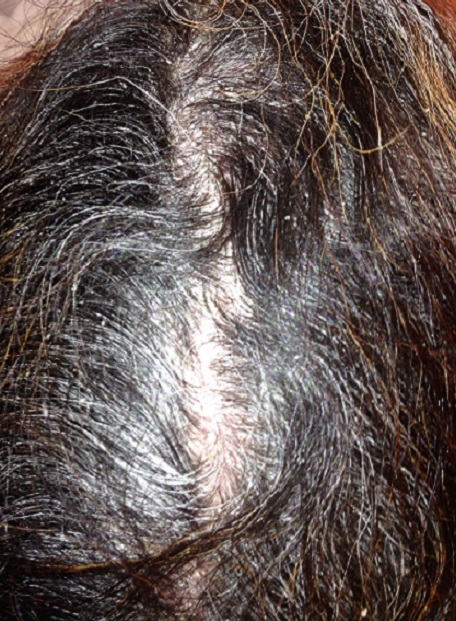
Psoriasis du cuir chevelu

Parmi les 200 cas de psoriasis, 46 cas colligés dans notre étude ont répondus aux critères d'inclusions. Il s'agissait de patients de sexe masculin dans 58,7% des cas avec un sexe – ratio H/F de 1,42, de sujets âgés entre 18 et 45 ans dans 45,7% des cas et de plus de 45 ans dans 41,3% des cas, alors que 13% étaient des enfants. Les formes cliniques étaient réparties comme suit: psoriasis vulgaire (Figure 1) dans 35 cas (76,1%), suivi du psoriasis arthropathique dans 6 cas (13%), le psoriasis érythrodermique dans 3 cas (6,5%) et la kératose palmo-plantaire psoriasique dans 2 cas (4,3%). L'atteinte des muqueuses était retrouvée dans seulement 9%, et celle du cuir chevelu (Figure 2) dans 72%. 71,7% avaient une SC atteinte comprise entre 25 et 50%, 21,7% avaient une surface comprise entre 50 et 70%, et seulement 6,5% avaient une SC supérieure à 70%.

La dose hebdomadaire du MTX variait de 10 à 30 mg, avec une dose moyenne de 25 mg prescrite chez 58,7%. Le MTX était associé dans 82,7% à l'acide folinique et aux dermocorticoïdes (DC), dans 15,2% aux DC seuls et dans seulement 2,2% le MTX était prescrit en association avec l'acide folinique et le calcipotriol. La durée moyenne de traitement était de 16,19 mois, avec une durée minimale de 6 mois et maximale de 24 mois. L’évolution était marquée par une rémission complète dans 50% des cas, avec un maximum de réponse obtenu à 24 mois. Les effets secondaires étaient essentiellement à type d'intolérance digestive, cytolyse et insuffisance rénale notés dans seulement 3 cas.

## Discussion

Nous avons mené une étude retro-prospective, sur une période de 4 ans, dont laquelle, on a évalué la place du méthotrexate dans le traitement du psoriasis dans notre contexte marocain. Notre étude est particulière, par la bonne réponse de nos patients, la rareté des effets secondaires, la bonne tolérance, témoignant ainsi de notre expérience dans la gestion du MTX.

Le psoriasis est une maladie chronique auto-immune, multifactorielle impliquant une interaction complexe entre des facteurs génétiques et environnementaux. En effet, les principales caractéristiques du psoriasis sont la prolifération et la différenciation anormale des kératinocytes, l′infiltration de l′épiderme par des cellules inflammatoires et enfin, des changements vasculaires. L′interaction entre les différents types cellulaires tels que les cellules épidermiques, les cellules impliquées dans la réponse immunitaire (les cellules T et les cellules présentatrices d′antigènes) et celles du système vasculaire (les cellules endothéliales) semble jouer un rôle dans la pathogenèse de la maladie, mais le défaut primaire responsable de cette maladie reste encore non identifié [4, 5]. La prévalence du psoriasis varie considérablement d'une région à une autre. Aux États-Unis, le psoriasis affecte environ 2% à 4,6% de la population [6]. Au Maroc, cette prévalence est de 1.79 / 1000 habitants soit une incidence annuelle de 1 à 2% [7]. Le psoriasis a une distribution bimodale caractéristique, la majorité des cas (environ 75%) ont un début avant l′âge de 40 ans, avec une association fréquente de l′antigène d′histocompatibilité (HLA)-Cw6 [7]. Nos données rejoignent ceux de la littérature, vu que presque la moitie de nos patients avait un âge inferieur à 45 ans.

Le psoriasis atteint la population générale sans prédilection de sexe [7], cependant dans notre série de psoriasis modéré à sévère, on a trouvé une prédominance masculine, en rapport probablement avec le retard de consultation des hommes au stade précoce de la maladie, ou probablement avec la prédisposition du sexe masculin aux formes graves du psoriasis. Le psoriasis se présente sous différentes formes, seuls les formes graves ou étendue nécessitent un traitement systémique, à savoir le psoriasis vulgaire atteignant plus de 25% de la surface corporelle, le psoriasis arthropatique,érythrodermique et la kératodermie palmo-plantaire psoriasique. L'arsenal thérapeutique du psoriasis est en perpétuelle progression surtout en matière de biothérapie. La prise en charge commence d'abord par des mesures générales, à savoir l’éviction des facteurs aggravants comme le stress, les infections surtout streptococciques, la consommation excessive d'alcool, de tabac ainsi que certains médicaments inducteurs [8]. Les traitements locaux sont adoptés pour les formes légères de psoriasis, c′est-à-dire lorsque la surface corporelle atteinte est inférieure à 15% ou en associations avec d'autres traitements systémiques locaux. Ils incluent les dermocorticoïdes, les analogues de la vitamine D3, un rétinoïde par voie locale, l′acide salicylique et les émollients [9]. Pour les formes plus sévères et résistantes aux traitements locaux, une photothérapie (UVB, PUVA) ou une thérapie systémique avec des rétinoïdes, méthotrexate, cyclosporine, voir des agents biologiques (L'infliximab:Rémicade^®^) sont envisagées [9, 10].

Depuis 1953, le méthotrexate a été utilisée aux Etats-Unis comme traitement antinéoplasique. Et depuis les années 1980, vu ses propriétés antiprolifératives et immunosuppressives, le méthotrexate a été utilisé pour traiter les grossesses ectopiques, mais aussi des pathologies inflammatoires comme la polyarthrite rhumatoïde et le psoriasis [11]. Le méthotrexate est un analogue de l′acide folique, cofacteur essentiel dans la synthèse de précurseurs de l′acide désoxyribonucléique (ADN) et de l′acide ribonucléique (ARN). Pour être actif, l′acide folique doit être réduit en tétrahydrofolates par une enzyme: la dihydrofolate réductase (DHFR). Le méthotrexate inhibe ainsi la DHFR par compétition avec I′acide folique, interrompant la synthèse de l'ADN et de l'ARN. II est donc essentiellement toxique pour les tissus à renouvellement rapide comme I’ épithélium intestinal, la moelle osseuse et les tissus néoplasiques [11–13].

Le méthotrexate (MTX) reste le pilier du traitement du psoriasis modéré à sévère, depuis sa première utilisation il y'a près d′un demi-siècle. Au fil des ans, son efficacité, son faible coût, la facilité relative de son administration, ont contribué à faire du MTX; le médicament de choix dans la gestion de psoriasis modéré à sévère. Une étude menée au service de Dermatologie de Casablanca sur une période de 15 ans (janvier 1991- décembre 2004), 2 013 patients atteints de psoriasis ont été colligés au service dont 458 patients hospitalisés pour formes graves. Parmi les 458 patients, 77 cas (16,8%) étaient traités par méthotrexate. La dose hebdomadaire du méthotrexate variait de 10 à 25 mg en IM. La durée d’évolution sous traitement était en moyenne de 78 mois. L’évolution était favorable dans 53,2% des cas, avec une rémission partielle dans 22% des cas. L'aggravation était notée dans 16,8% des cas [14].

Une autre étude menée en Tunisie, de janvier 2002 à décembre 2009. 21 patients atteints de psoriasis sévère et mis sous méthotrexate ont été recensés. La rémission était totale dans 62% des cas et partielle dans 28,5% des cas [15]. Dans notre série le recours au MTX était nécessaire et ceci après échec des dermocorticoïdes dans 39 cas, le calcipotriol dans 5 cas et la photothérapie dans 2 cas. La dose hebdomadaire du MTX variait de 10 à 30 mg. La durée moyenne de traitement était de 16,19 mois. Le suivi des patients se faisait grâce à un bilan biologique et au fibroscann. La supplémentation par l'acide folique était réalisée chez 82,7%. Son efficacité a été constatée à partir du troisième mois, 50% des patients étaient en rémission complète après 24 mois de traitement. Les effets secondaires étaient rarement rencontrés dans notre étude, probablement grâce au suivi régulier des patients et la supplémentation en acide folique. Par ailleurs le faible prix du MTX a élargit les indications de sa prescription, surtout chez les patients avec un niveau socio-économique bas. En effet, le calcul du cout du traitement sur un an chez un patient de 60 Kg, sans prendre en considération le cout du bilan, a objectivé que le MTX est114 fois moins cher que l'infliximab. Le MTX reste pour nous un traitement de choix, qu'on a l'habitude de prescrire: il est sur, efficace, de faible cout et bien toléré. Les biothérapies sont aussi efficace que le MTX avec moins d'effets secondaires, cependant leur coût reste élevé, d'autant plus qu'ils ne sont pas remboursé par la sécurité sociale. Dans notre contexte, le MTX reste le traitement de première ligne pour le psoriasis modéré à sévère.

## Conclusion

Le MTX a été le traitement standard du psoriasis modérée à sévère depuis plus de cinq décennies. Son efficacité est indiscutable, et il est important de reconnaître que le MTX continuera à être le pilier du traitement du psoriasis, en particulier dans les pays en développement, en raison de son rapport coût-efficacité
